# Rice OsRH58, a chloroplast DEAD-box RNA helicase, improves salt or drought stress tolerance in *Arabidopsis* by affecting chloroplast translation

**DOI:** 10.1186/s12870-018-1623-8

**Published:** 2019-01-09

**Authors:** Ghazala Nawaz, Hunseung Kang

**Affiliations:** 1 Department of Applied Biology, College of Agriculture and Life Sciences, Chonnam National University, 77 Yongbong-ro, Buk-gu, Gwangju, 61186 South Korea; 2 Department of Botany, Kohat University of Science and Technology, Indus Highway Kohat, Kohat, Khyber Pakhtunkhwa 26000 Pakistan

**Keywords:** Abiotic stress, Arabidopsis, Chloroplast, DEAD-box RH, Rice, RNA helicase

## Abstract

**Background:**

Despite increasing characterization of DEAD-box RNA helicases (RHs) in chloroplast gene expression regulation at posttranscriptional levels in plants, their functional roles in growth responses of crops, including rice (*Oryza sativa*), to abiotic stresses are yet to be characterized. In this study, rice OsRH58 (LOC_Os01g73900), a chloroplast-localized DEAD-box RH, was characterized for its expression patterns upon stress treatment and its functional roles using transgenic *Arabidopsis* plants under normal and abiotic stress conditions.

**Results:**

Chloroplast localization of OsRH58 was confirmed by analyzing the expression of OsRH58-GFP fusion proteins in tobacco leaves. Expression of *OsRH58* in rice was up-regulated by salt, drought, or heat stress, whereas its expression was decreased by cold, UV, or ABA treatment. The OsRH58-expressing *Arabidopsis* plants were taller and had more seeds than the wild type under favorable conditions. The transgenic plants displayed faster seed germination, better seedling growth, and a higher survival rate than the wild type under high salt or drought stress. Importantly, levels of several chloroplast proteins were increased in the transgenic plants under salt or dehydration stress. Notably, OsRH58 harbored RNA chaperone activity.

**Conclusions:**

These findings suggest that the chloroplast-transported OsRH58 possessing RNA chaperone activity confers stress tolerance by increasing translation of chloroplast mRNAs.

**Electronic supplementary material:**

The online version of this article (10.1186/s12870-018-1623-8) contains supplementary material, which is available to authorized users.

## Background

Plants are sessile organisms facing diverse environmental stresses, such as temperature shock, high salinity, drought, and UV. These stresses severely affect plant growth and productivity. Photosynthesis in chloroplasts is an essential cellular process that is greatly affected by environmental stresses [1, 2]. To withstand environmental stresses, plants need to regulate cellular metabolic processes, including photosynthesis, that are crucial for plant growth and productivity under abiotic stresses [3, 4]. Chloroplast gene expression is regulated at the level of pre-RNA processing, base editing, splicing, decay, and translation [5–7]. Although fewer than 150 proteins are encoded by the chloroplast genome, the chloroplast contains more than 3000 proteins that are encoded by the nucleus genome and transported into the chloroplast. Therefore, inter-communications between the chloroplast and the nucleus via bidirectional signaling are required for fine-tuned gene expression in both organelles [8–13].

Many RNA-binding proteins (RBPs), including DEAD-box RNA helicases (RHs), are transported to the chloroplast. RNA helicases are proteins that can unwind RNAs and remodel structured RNAs [14, 15]. Many previous reports have demonstrated that DEAD-box RHs are cellular molecules important for plant growth and fitness in harsh environments (reviewed in [16, 17]). Although many DEAD-box RHs in the nucleus have been characterized in plants, particularly in *Arabidopsis thaliana*, functions of the many chloroplast-localized DEAD-box RHs in crops, including rice, are yet to be characterized. Chloroplast-localized AtRH3, HVD1, and BrRH22 have been shown to increase plant growth under drought or salinity stress [18–20]. Chloroplast-localized AtRH3 or mitochondria-localized ABO6 regulate auxin or ABA signaling [19, 21, 22]. A genome-wide search for the putative chloroplast DEAD-box RHs in *Arabidopsis,* maize (*Zea mays*), rice (*Oryza sativa*), and wheat (*Triticum aestivum*) has revealed that approximately 7 to 12 nucleus-encoded DEAD-box RHs contain potential chloroplast-targeting signal peptides [17].

Rice is an economically important cereal crop, the productivity of which is severely diminished by diverse abiotic stresses [23]. Given the possibility of using chloroplast-localized DEAD-box RHs to engineer stress-tolerant crops, it is important to determine the roles of chloroplast-localized DEAD-box RHs in rice under stress conditions. Despite the increasing notifications on the potential roles of chloroplast-localized DEAD-box RHs in plant fitness under abiotic stresses, the roles of DEAD-box RHs in rice chloroplasts are largely unknown. To date, only a few RHs, such as OsABP and OsRH53, have been characterized in rice’s response to abiotic stresses [24, 25]. In this study, we characterized the function of a chloroplast-localized OsRH58 (LOC_Os01g73900) in abiotic stress response by using transgenic *Arabidopsis* plants.

## Results

### Subcellular localization and stress-responsive expression patterns of OsRH58 in rice

**Fig. 1:**
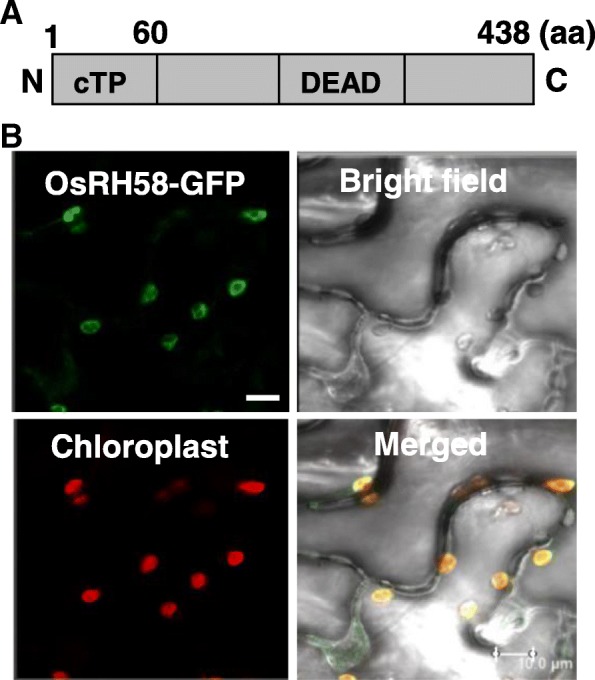
Domain structures and subcellular localization of OsRH58. **a** Schematic representation of the domain structures of OsRH58. The 60 amino acid (aa)-long chloroplast transit peptide (cTP) at the N-terminal end and the Asp-Glu-Ala-Asp (DEAD) motif are shown. **b** The GFP signals arising from the OsRH58-GFP fusion proteins expressed in tobacco leaves were detected using a confocal microscope. Red signals represent chloroplast auto-fluorescence. Scale bar = 10 μm

OsRH58 was identified as a putative chloroplast-localized DEAD-box RH in a previous study [17]. It consists of 438 amino acid residues and contains a 60 amino acid-long putative chloroplast transit peptide (cTP) at the N-terminal end (Fig. 1a). To confirm whether OsRH58 is localized to chloroplasts, the *Agrobacterium* containing the OsRH58-GFP vector was infiltrated into tobacco leaves, and the transiently expressed OsRH58-GFP fusion proteins were observed using a confocal microscope. GFP signals and chloroplast auto fluorescence were merged together (Fig. 1b), confirming chloroplast localization of the OsRH58 protein.

**Fig. 2:**
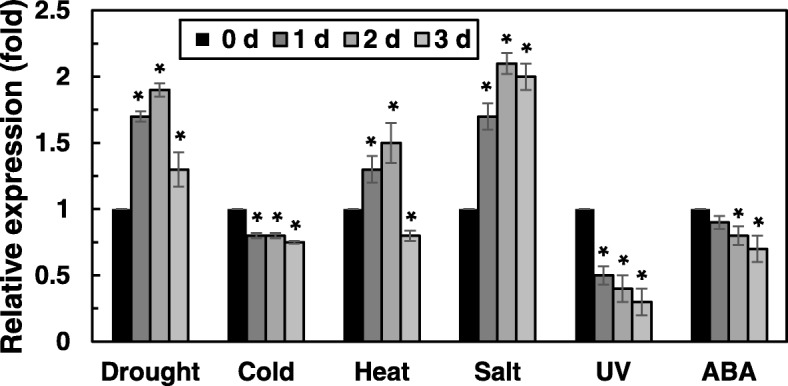
Expression of *OsRH58* in rice under abiotic stress treatment. Three-week-old rice seedlings were subjected to drought, 100 μM ABA, cold (10 °C), UV (5 watt), salt (150 mM NaCl), or heat (45 °C), and *OsRH58* levels were measured by qRT-PCR. The mean and standard error were obtained from three biological replicates, and asterisks indicate statistically significant differences (*P* ≤ 0.05)

To analyze the stress-response expression patterns of *OsRH58* in rice, different abiotic stresses were applied to rice seedlings, and *OsRH58* levels were measured by quantitative RT-PCR analysis. The degree and adequacy of stress treatment in rice was shown in the previous report [25]. Drought, salt, or heat stress increased the expression of *OsRH58* up to two-fold. Cold stress or ABA marginally decreased *OsRH58* levels, whereas UV stress significantly decreased the expression of *OsRH58* (Fig. 2).

### OsRH58 increases the growth and seed yield of *Arabidopsis* plants under normal conditions

**Fig. 3:**
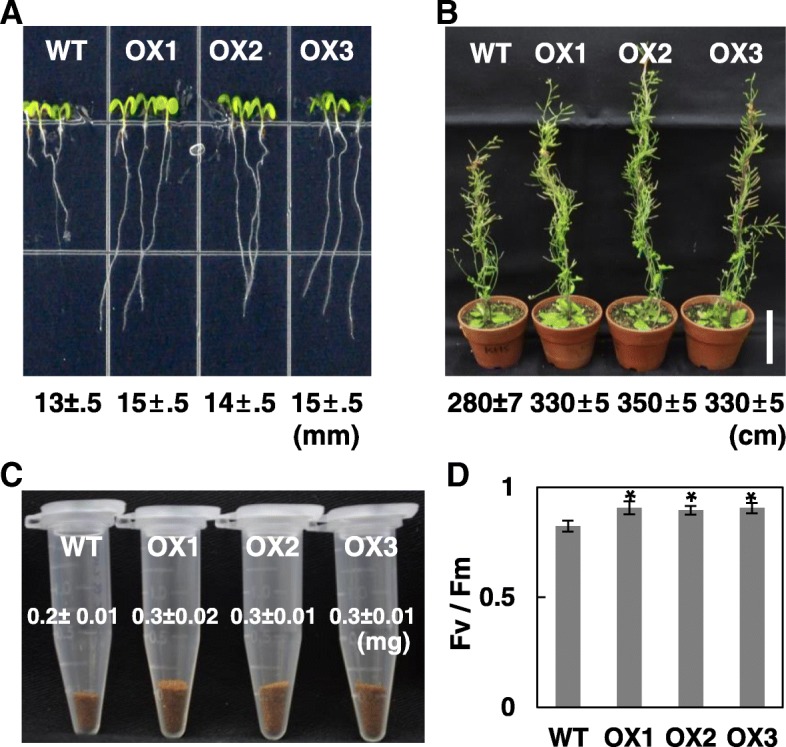
OsRH58 improves *Arabidopsis* growth under normal conditions. **a** Root length of the wild type (WT) and OsRH58-expressing transgenic *Arabidopsis* plants (OX1, OX2, and OX3) was measured on MS medium 14 days after germination. **b** Plant height and **c** seed yield were measured at maturity. **d** The maximum quantum yield (Fv/Fm) of PSII was measured 4 weeks after germination. The mean and standard error were obtained from three biological replicates, and asterisks indicate statistically significant differences (*P* ≤ 0.05)

Given that abiotic stresses modulate *OsRH58* expression, it is interesting to determine the functional roles of OsRH58 in stress responses. Although we need to analyze the knockout mutant of OsRH58 to fully understand its function in rice, in this study we evaluated the function of OsRH58 using transgenic *Arabidopsis* plants that express OsRH58 because the OsRH58 mutant was not available. The expression of *OsRH58* in the three independent homozygous lines, which displayed similar phenotypes, was confirmed by RT-PCR (Additional file 1 a). The wild type and transgenic *Arabidopsis* seeds germinated equally well without differences on normal MS medium (Additional file 1 b). However, the transgenic plants had slightly longer roots than did the wild type (Fig. 3a). At maturity, the transgenic *Arabidopsis* plants were taller and had more seeds than the wild type (Fig. 3b and c), and the maximum quantum yields of PSII (Fv/Fm) of the wild type and transgenic *Arabidopsis* plants were approximately 0.82 and 0.9, respectively (Fig. 3d). These results indicate that OsRH58 promotes plant growth and seed yield under normal conditions.

### OsRH58 has positive effects on seed germination of *Arabidopsis* under salinity or drought stress

**Fig. 4:**
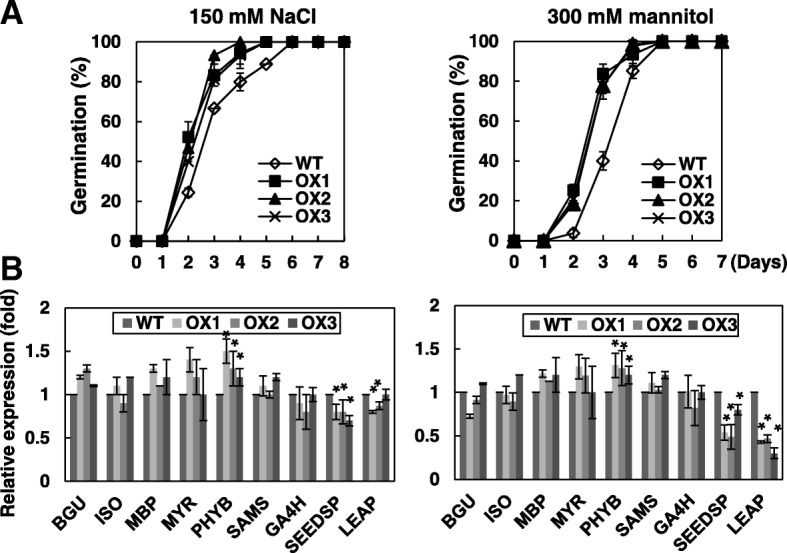
OsRH58 accelerates seed germination under salt or dehydration stress. **a** Germination rates of the wild type (WT) and OsRH58-expressing transgenic *Arabidopsis* plants (OX1, OX2, and OX3) were scored on MS medium supplemented with 150 mM NaCl or 300 mM Mannitol. **b** Transcript levels of each gene were measured at the second day by qRT-PC. The mean and standard error were obtained from three biological replicates, and asterisks indicate statistically significant differences (*P* ≤ 0.05)

The role of OsRH58 in seed germination under stress was evaluated by comparing germination rates of the wild type and transgenic seeds under various abiotic stress treatments. Approximately 50 and 85% of transgenic seeds germinated on the second and third day, respectively, on MS medium containing 150 mM NaCl, whereas approximately 25 and 65% of wild type seeds germinated on the same days (Fig. 4a). Given 300 mM mannitol treatment, approximately 25 and 80% of transgenic seeds germinated on the second and third day, respectively, whereas only 3 and 40% of wild type seeds germinated on the same days (Fig. 4a). In contrast, the wild type and transgenic seeds germinated without noticeable differences under cold stress or ABA treatment (Additional file 1 b).

To understand how OsRH58 affects seed germination under high salt or dehydration stress, we examined expression levels of germination-responsive genes, including the positive effectors of germination, such as β-glucosidase (BGU), GA4 homolog (GA4H), isocitrate lyase (ISO), myrosinase (MYR), myrosinase-binding protein (MBP), phytochrome B (PHYB), and S-adenosyl methionine synthetase (SAM) [26, 27], and the negative effectors of seed germination, such as seed storage protein (SEEDSP) and LEA protein (LEAP) [27]. Results showed that the level of a positive effector *PHYB* was marginally increased in transgenic plants under salinity or dehydration stress, whereas the transgenic *Arabidopsis* plants had much lower levels of negative effectors (*SEEDSP* and *LEAP*) than the wild type (Fig. 4b). These results suggest that the better germination of the transgenic seeds under salinity or dehydration stress partly results from the modulation of these effectors of germination by OsRH58.

### OsRH58-expressing transgenic plants are tolerant to salinity or drought stress

**Fig. 5:**
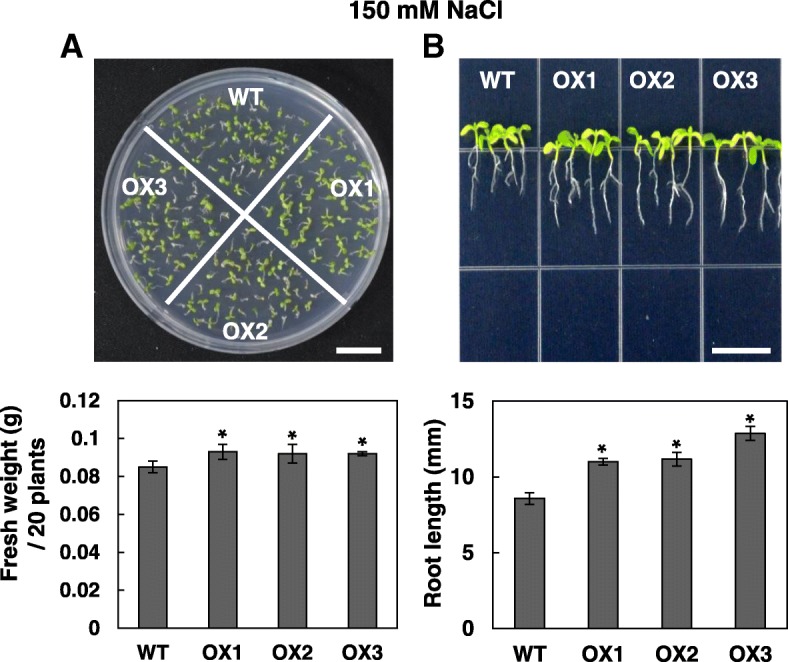
OsRH58-expressing transgenic plants are tolerant against salinity stress. The wild type (WT) and OsRH58-expressing transgenic *Arabidopsis* plants (OX1, OX2, and OX3) were grown on MS medium containing 150 mM NaCl, and (**a**) seedling fresh weight and (**b**) root length were measured after 3 weeks and 2 weeks, respectively. The mean and standard error were obtained from three biological replicates, and asterisks indicate statistically significant differences (*P* ≤ 0.05). Scale bar = 1 cm

**Fig. 6:**
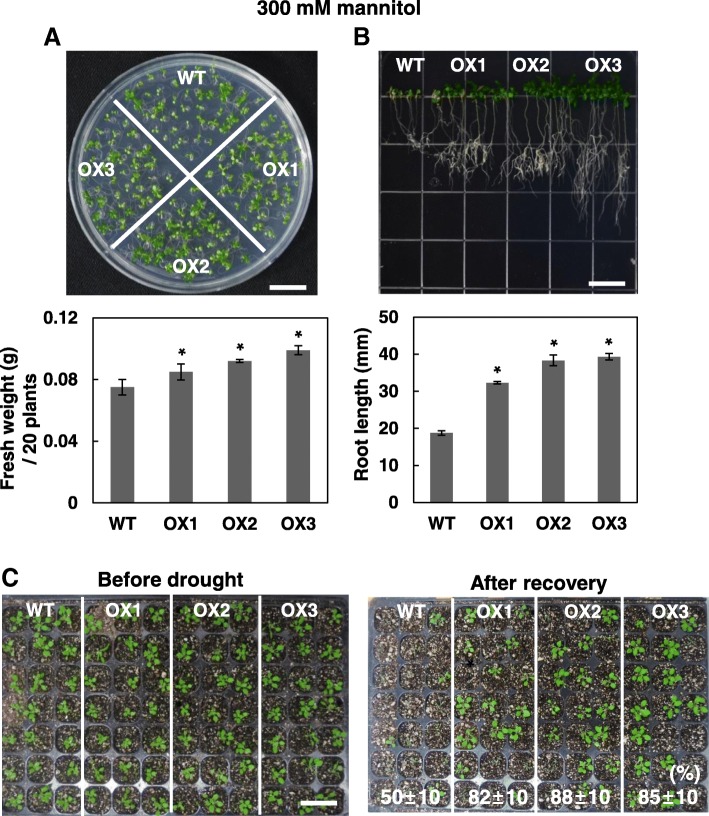
OsRH58-expressing transgenic plants are tolerant against drought stress. The wild type (WT) and OsRH58-expressing transgenic *Arabidopsis* plants (OX1, OX2, and OX3) were grown on MS medium containing 300 mM mannitol, and (**a**) seedling fresh weight and (**b**) root length were measured after 3 weeks and 2 weeks, respectively. **c** Two-week-old seedlings in soil were unwatered for 4 days, and survival rates were scored at 1 week after recovery. The mean and standard error were obtained from three biological replicates, and asterisks indicate statistically significant differences (*P* ≤ 0.05). Scale bar = 1 cm

When the wild type and OsRH58-expressing *Arabidopsis* plants were grown under normal, cold, or ABA conditions, their seedling growth was similar to each other (Additional file 2). In contrast, the transgenic plants tolerated salinity or drought stress much better. The transgenic *Arabidopsis* plantlets showed marginally better seedling growth and noticeably better root growth than the wild type on MS medium containing 150 mM NaCl (Fig. 5). When subjected to dehydration stress on MS medium with 300 mM mannitol, the fresh weight and root length of the OsRH58-expressing transgenic *Arabidopsis* plants were significantly higher than those of the wild type (Fig. 6a and b). To further evaluate the role of OsRH58 in drought stress tolerance, survival rates of the wild type and OsRH58-expressing transgenic seedlings grown in soil were measured after recovering from four days of drought stress treatment. Approximately 85% of the transgenic seedlings survived after recovery, whereas approximately 50% of the wild type seedlings survived (Fig. 6c). These results indicate that OsRH58 confers salt or drought tolerance but not cold tolerance.

### OsRH58 increases chloroplast protein levels under salt or dehydration stress

**Fig. 7:**
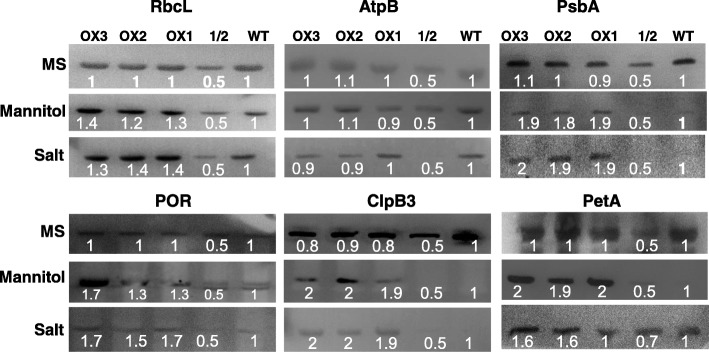
OsRH58 affects the levels of chloroplast proteins under salt or dehydration stress. Total proteins were extracted from 2-week-old wild type (WT) and OsRH58-expressing transgenic *Arabidopsis* plants (OX1, OX2, and OX3) grown on MS medium containing 300 mM mannitol or 150 mM NaCl. Twenty to thirty micrograms of total proteins were separated on SDS-12% PAGE, and the proteins were detected with the antibody raised against each protein. The intensities of each band were calculated, and the mean and standard error were obtained from three biological replicates. 1/2, half amount of WT protein

The next important question is how OsRH58 confers stress tolerance. Because OsRH58 is localized in chloroplasts, it should be involved in chloroplast RNA metabolism. We, therefore, first analyzed the efficiency of the splicing of chloroplast introns under normal, salt, or dehydration stress conditions. Notably, the splicing efficiencies of chloroplast introns in the wild type and OsRH58-expressing transgenic *Arabidopsis* plants were comparable to each other under normal, salinity, or dehydration conditions (Additional file 3). We next assessed by immunoblot analysis whether OsRH58 affects translation of chloroplast transcripts. In this study, the levels of casein lytic proteinase B3 (ClpB3), D1 protein of PSII (PsbA), cytochrome f protein (PetA), protochlorophilide oxidoreductase (POR), rubisco large subunit (RbcL), and beta subunit of ATP synthase (AtpB) were examined, due to the availability of these antibodies in our laboratory. Equal loading of total proteins in each lane on the gel was verified by Ponceau-S staining (Additional file 4). The results showed that the OsRH58-expressing transgenic plants accumulated much larger amounts of ClpB3, PsbA, PetA, POR, and RbcL than did the wild type under salt or dehydration stress (Fig. 7). In comparison, the wild type and transgenic plants had similar amounts of proteins when tested under normal conditions (Fig. 7). Altogether, these data suggest that OsRH58 confers stress tolerance by influencing the translation of chloroplast mRNAs.

### OsRH58 harbors RNA chaperon activity

**Fig. 8:**
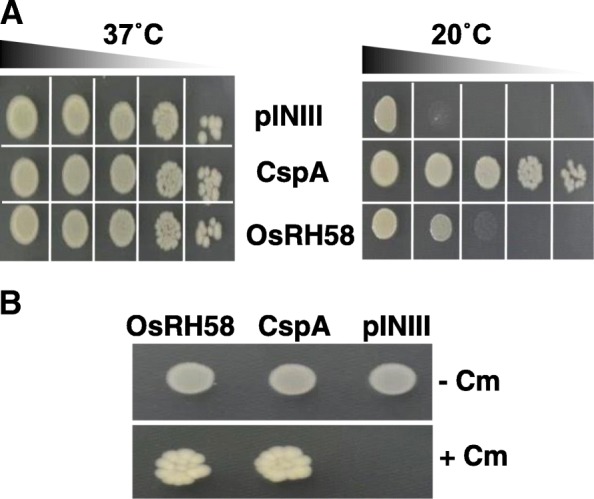
OsRH58 possesses RNA chaperone activity. **a** The complementation ability of OsRH58 in *Escherichia coli* BX04 mutant cells was evaluated by incubating the diluted cell cultures at 20 °C. **b** The transcription anti-termination activity of OsRH58 in *E. coli* RL211 mutant cells was evaluated by growing the cells on LB agar medium with (+) or without (−) chloramphenicol. The pINIII vector and pINIII-CspA were used as negative and positive control, respectively

The next important question is how OsRH58 affects translation of chloroplast genes. Considering that many DEAD-box RHs participate in translation control by functioning as RNA chaperones [28–30], it is possible that OsRH58 participates in the translation of chloroplast mRNAs through RNA chaperone activity. We tested this possibility by analyzing the RNA chaperone activity of OsRH58. We, first, evaluated the complementation capability of OsRH58 in *E. coli* mutant BX04 cell that is sensitive to cold shock because of the lack of RNA chaperones [31]. The BX04 cells harboring OsRH58, CspA (used as a positive control), or pINIII (used as a negative control) grew similarly at a normal temperature (37 °C). In contrast, the BX04 cells harboring OsRH58 or CspA grew well at a low temperature (20 °C), whereas the growth of the pINIII-harboring BX04 cells was poor at 20 °C (Fig. 8a). We next evaluated the base pairs-breaking ability of OsRH58 in *E. coli* RL211 cells in which the chloramphenicol (Cm)-resistant gene is expressed if base pairs on the RNA are disrupted in *trpL* terminator located upstream of the Cm-resistant gene [32]. The RL211 cells having either OsRH58 or CspA grew well on LB medium containing chloramphenicol, whereas the RL211cells possessing pINIII did not grow (Fig. 8). These results confirm that OsRH58 harbors RNA chaperone activity.

## Discussion

Despite the potential role of chloroplast-localized DEAD-box RHs in plant adaptation to abiotic stresses, reports characterizing the chloroplast localization and function of DEAD-box RHs in crop species are few. The current results show that the chloroplast-localized rice OsRH58 can improve seed germination, seedling growth, and plant vigor under both normal and stress conditions. Notably, OsRH58 increased seed yield in *Arabidopsis* under normal conditions (Fig. 3). It would be valuable to further evaluate whether OsRH58 can be used to increase seed yield in rice under normal growth conditions. Constitutive expression of OsRH58 in *Arabidopsis* accelerated seed germination and increased seedling growth and survival when subjected to salt or dehydration stress (Figs. 4, 5, and 6). Because the expression of *OsRH58* increased in rice under salt or drought stress (Fig. 2), it is likely that OsRH58 performs a similar positive role in rice under salt or drought stress. It would be valuable to evaluate whether OsRH58 can be used to engineer salt- or drought-tolerant rice cultivars. A recent study has also demonstrated that expression of BrRH22, a chloroplast-transported DEAD-box RH in cabbage (*Brassica rapa*), whose transcript level is markedly up-regulated by salt, drought, cold, or heat stress, improves germination and plant vigor in transgenic *Arabidopsis* under salt or drought stress [25]. In contrast, expression of nuclear OsRH53, whose transcript level was decreased in rice by salt or drought stress, had a negative effect on the germination and growth of *Arabidopsis* under salt or dehydration stress [25]. These results suggest that expression patterns of DEAD-box RHs in crops modulated by abiotic stresses are closely correlated with their stress-tolerance functions. However, it was also noted that, although the expression of rice *OsRH58* and cabbage *BrRH22* was increased by cold stress, the OsRH58- or BrRH22-expressing transgenic *Arabidopsis* showed no tolerance to cold stress (20; Additional file 2). These results suggest that more functional analysis of DEAD-box RHs is required in crop species to fully understand their roles in stress tolerance. Moreover, recent systematic analyses of the organelle-targeted or potential DEAD-box RHs in crops such as rice, maize, wheat, and tomato highlighted the number and proposed roles of DEAD-box RHs in growth, development, and stress responses in crops [17, 33]. The functions and significance of these DEAD-box RHs in crops await further characterization.

It is important to understand the cellular roles of chloroplast DEAD-box RHs. Considering that RNA metabolic processes, including intron splicing, pre-RNA processing, editing, and translation, are main regulatory steps in chloroplast gene expression [5, 7], DEAD-box RHs should be involved in these cellular processes. Previous studies clearly demonstrated that the chloroplast-localized DEAD-box RHs, ISE2 and RH3, are involved in the splicing of chloroplast introns in *Arabidopsis* [19, 21, 34]. A recent overview emphasizes the roles of DEAD-box RHs in rRNA processing during plant growth and stress responses [35]. Our current results showed that OsRH58 has no role in the splicing of chloroplast introns (Additional file 3) but influences translation of several chloroplast transcripts (Fig. 7). Several RHs have been known to participate in translation control [36]. The chloroplast-localized *Arabidopsis* DEAD-box AtRH22 and its cabbage ortholog BrRH22 are involved in the assembly of 50S ribosomes and translation of chloroplast transcripts [20, 37]. In addition, the chloroplast-targeted ISE2 and RH50 play essential roles in chloroplast rRNA maturation in *Arabidopsis* [38, 39]. These current and previous findings altogether suggest the crucial role of DEAD-box RHs in translation of chloroplast mRNAs. Notably, alteration in chloroplast translation is an adaptive response of plants to abiotic stress [40]. Further studies are required to fully understand how OsRH58 influences translation of chloroplast mRNA under salt or drought stress.

Discovering the mechanistic role of OsRH58 in chloroplast translation is a remaining critical question. Because RNA molecules are prone to adapt alternative non-functional structures under stress, resolving these misfolded structures are essential for proper function of RNAs. RNA chaperones are RBPs that can rearrange the structure of diverse RNA molecules [41–43]. The importance of RHs as RNA chaperones in plant stress responses has been emerging (reviewed in [29, 30, 44, 45]). Notably, the bacterial and yeast DEAD-box RHs act as RNA chaperones in the process of intron splicing [46–50]. The *Arabidopsis* RH3 participates in the splicing of chloroplast introns via its RNA chaperone activity [19]. Our findings show that OsRH58 possesses RNA chaperone activity (Fig. 8), which influences the translation of several chloroplast mRNAs. Importantly, RNA chaperones are known to bind to RNA targets with a low sequence preference [28]. It is likely that OsRH58 binds to diverse chloroplast mRNAs, and its RNA chaperone activity rearranges the RNA structures, which affects the translation of several target mRNAs such as POR, RbcL, ClpB3, PsbA, and PetA (Fig. 7). Because OsRH58 does not affect the levels of chloroplast proteins under normal conditions (Fig. 7), further studies are required to find out whether the structures of chloroplast mRNAs change in ways that depend on specific stress conditions and to fully understand how OsRH58 influences translation of chloroplast mRNA under salt or drought stress.

## Conclusions

Our results highlight the importance of chloroplast-localized OsRH58 in plant growth and vigor under abiotic stresses. Although it is clear that OsRH58 helps to improve the germination and growth of *Arabidopsis* under salt or drought stress, the stress-responsive role of OsRH58 in rice remains to be investigated. In particular, it would be interesting to determine whether chloroplast-located OsRH58 affects the expression or activity of ROS scavenging enzymes that are deeply associated with stress tolerance in plants. Furthermore, evaluation of the functions of as yet uncharacterized chloroplast-transported DEAD-box RHs and their cellular roles in stress response awaits further investigation. Considering the potential applications of DEAD-box RHs in developing stress-tolerant crops, it would be of value to engineer DEAD-box RHs using a CRISPR-Cas9 genome editing technology to introduce or delete specific DEAD-box RHs whose expression are up- or down-regulated by abiotic stresses.

## Methods

### Determination of subcellular localization of OsRH58

OsRH58 was identified in a previous study as a putative chloroplast-localized DEAD-box RH that contains a potential chloroplast transit peptide (cTP) at the N-terminal end [17]. To confirm the chloroplast localization of OsRH58, *Agrobacterium tumefaciens* GV3101 harboring the CsV-OsRH58-GFP vector was infiltrated into tobacco leaves, and the GFP signals were examined using a laser scanning confocal microscope (Carl Zeiss Inc., Thornwood, NY, USA) with the excitation wavelength of 488 nm and the emission wavelength of 505–545 nm. Chloroplast auto-fluorescence was examined with the excitation and emission wavelengths of 633 nm and 640–690 nm, respectively.

### Rice materials and stress treatment

The *A. thaliana* used in this study was Columbia-0 ecotype, which was obtained from the Arabidopsis Biological Resource Center (Columbus, OH, USA). Rice (Dongjin variety) seeds were obtained from the Crop Biotech Institute of Kyung Hee University (Suwon, Korea). Rice seeds were sown on Murashige and Skoog (MS) medium, and germinated seedlings were transferred to soil and kept growing in a growth room at 30 °C under 16/8 h light/dark cycles. Various abiotic stresses such as drought, salt, cold, or UV, and ABA were applied to four-week-old rice plantlets as previously described [25]. Briefly, watering was stopped for drought stress, and the pots were immersed in 300 mM NaCl solution for salt stress. Cold or heat stress was applied by growing the plants at 10 °C or 39 °C, respectively. The rice seedlings were grown under 3.0 W m^− 2^ UV light for UV stress, and 100 μM ABA solution was sprayed onto the plants.

### RNA extraction and real-time RT-PCR analysis

Plant materials were ground, and total RNA was extracted using a Plant RNeasy kit (Qiagen, Valencia, CA, USA). Real-time quantification of transcript levels was performed on a Rotor-Gene Q (Qiagen) using MG2X SYBR Green RT-PCR kit (Qiagen) as described [51]. Splicing efficiency was measured by qRT-PCR with primers to amplify exon/exon (spliced or mature) and intron/exon (unspliced or precursor) transcripts as described [52, 53]. Primers used in qRT-PCR are shown in Additional file 5. Action was used as a reference gene.

### Generation of OsRH58-expressing transgenic *Arabidopsis* plants

The cDNA encoding OsRH58 was amplified by RT-PCR and cloned into pCAMBIA1301 vector, which directs constitutive expression of OsRH58 under the cauliflower mosaic virus 35S promoter. Vacuum infiltration [54] using *Agrobacterium tumefaciens* GV3101 was used to transform *Arabidopsis*. Selection of transgenic *Arabidopsis* plants was carried out on MS medium supplemented with hygromycin (50 μg mL^− 1^) and carbenicillin (250 μg mL^− 1^). Homozygous T_4_ lines were obtained and used for subsequent analysis. To confirm the expression of *OsRH58* in transgenic *Arabidopsis* plants, RT-PCR was performed using the primers listed in Additional file 5.

### Seed germination and seedling growth assays under abiotic stresses

The wild type and transgenic seeds were sown on MS medium containing 1% sucrose. The plates were kept at 4 °C in the dark for three days for stratification and then were incubated at 25 °C under 16/8 h light/dark cycles. The effects of high salt or drought stress on seed germination were evaluated by sowing the seeds on MS medium containing either 150–200 mM NaCl or 300–450 mM mannitol, respectively. The effects of cold stress on seed germination were evaluated by incubating the plates at 10 °C, and ABA effects on seed germination were analyzed by sowing the seeds on MS medium with 1–5 μM ABA. Germination rate was scored by observing radicle protrusion. To evaluate the effects of different stresses on root and seedling growth, 3-day-old seedlings that were fully germinated on normal MS medium were transferred to MS plates supplemented with NaCl or mannitol, or incubated at low temperatures as described above. To measure root length, the MS plates were incubated vertically.

### Measurement of photosynthetic activity

Leaves of the wild type and transgenic plants were adapted to darkness for 20 min, and the maximum quantum yield of PSII (Fv/Fm) was measured using Handy PEA chlorophyll fluorimeter (Hansatech instruments Ltd., England) according to the manufacturer’s instruction.

### Protein extraction and immunoblot analysis

Plant materials were ground, and total proteins were extracted in a buffer (50 mM Tris-HCl, 100 mM DTT, pH 7.5, 1 mM EDTA, 250 mM sucrose, 5 mM leupeptin and 100 mM PMSF). A SDS-12% polyacrylamide gel electrophoresis was used to separate proteins, after which proteins on the gel were blotted to a polyvinylidene difluoride membrane. The primary antibody specific to each protein was incubated with the membrane. After binding the secondary antibody with horse radish peroxidase, chemiluminescence signals were detected with an image analyzer (GE Healthcare Life Sciences, USA). Ponceau-S solution containing 5% acetic acid and 0.1% Ponceau-S was used to stain the total proteins on the membrane.

### RNA chaperone activity assay

The cDNA encoding OsRH58 was amplified by RT-PCR and cloned into a pINIII vector. For a complementation assay in *Escherichia coli* BX04 mutant cells [31], the pINIII-OsRH58, pINIII-CspA, or pINIII vector was introduced into the cell. Serially diluted cultures of BX04 cells expressing each construct were spotted on Luria-Bertani (LB) agar plates, and the plates were incubated at 20 °C. Transcription anti-termination assay was performed by using the *E. coli* RL211 cells [32]. The pINIII-OsRH58, pINIII-CspA, or pINIII vector was introduced into the RL211 cells, and cultures of the transformants were spotted on LB plates containing tetracycline and with or without chloramphenicol (Cm). The plates were incubated at 37 °C.

### Statistical analysis

The differences in gene expression levels and growth parameters between the wild type and transgenic plants were compared by *t* test (*p* ≤ 0.05) using the SigmaPlot 10 program (Systat Software, Inc., San Jose, CA, USA).

## Additional files

Additional file 1: Confirmation and seed germination of transgenic plants under normal, cold, or ABA conditions. (A) Expression of *OsRH58* in three homozygous *Arabidopsi*s lines (OX1, OX2, and OX3) was confirmed by RT-PCR. Actin was used as a loading control. (B) Germination rates of the wild type (WT) and transgenic plants were scored on MS medium or MS medium supplemented with 1 μM ABA at normal temperatures, and on MS medium at 10 °C. (PDF 406 kb)

Additional file 2: Seedling growth of the wild type and transgenic plants under normal, cold, or ABA conditions. Growth of the wild type (WT) and OsRH58-expressing transgenic *Arabidopsis* plants (OX1,OX2, and OX3) was analyzed on MS medium or MS medium supplemented with 2 μM ABA at normal temperatures, and on MS medium at 10 °C. The mean and standard error of fresh weight were obtained from three biological replicates. (PDF 408 kb)

Additional file 3: Splicing efficiency of chloroplast intron-containing genes. Total RNA was extracted from 2-week-old wild type (WT) and OsRH58-expressing transgenic *Arabidopsis* plants (OX1,OX2, and OX3) grown on MS medium or MS medium supplemented with 150 mM NaCl or 300 mM mannitol, and the levels of unspliced and spliced transcripts of each gene were determined by real-time RT-PCR. The mean and standard error were obtained from three biological replicates. (PDF 405 kb)

Additional file 4: SDS-PAGE gels showing total proteins in each sample. Total proteins were extracted from 2-week-old wild type (WT) and OsRH58-expressing transgenic *Arabidopsis* plants (OX1,OX2, and OX3) grown on MS medium or MS medium supplemented with 150 mM NaCl or 300 mM mannitol, and the proteins were separated on SDS-12% PAGE gel, transferred to membrane, and stained with a Ponceau-S. 1/2, half amount of WT protein. (PDF 408 kb)

Additional file 5: List of primers used in RT-PCR and quantitate real-time RT-PCR analysis. (PDF 403 kb)

## Abbreviations

DEAD: asp-glu-ala-asp
RBP: RNA-binding protein
RH: RNA helicase
