# Supplementary figures and images for: Rice OsRH58, a chloroplast DEAD-box RNA helicase, improves salt or drought stress tolerance in Arabidopsis by affecting chloroplast translation
Source: BMC Plant Biol. 2019 Jan 9;19:17. doi: 10.1186/s12870-018-1623-8 (PMC6327599; doi:10.1186/s12870-018-1623-8)

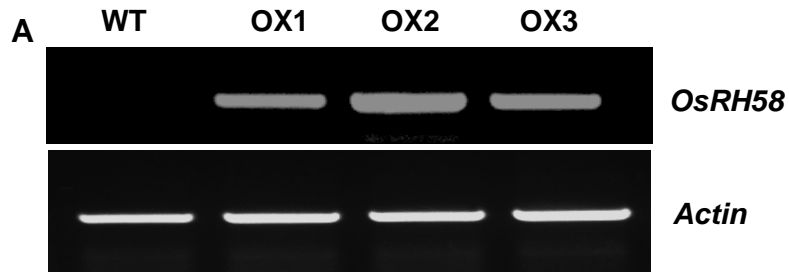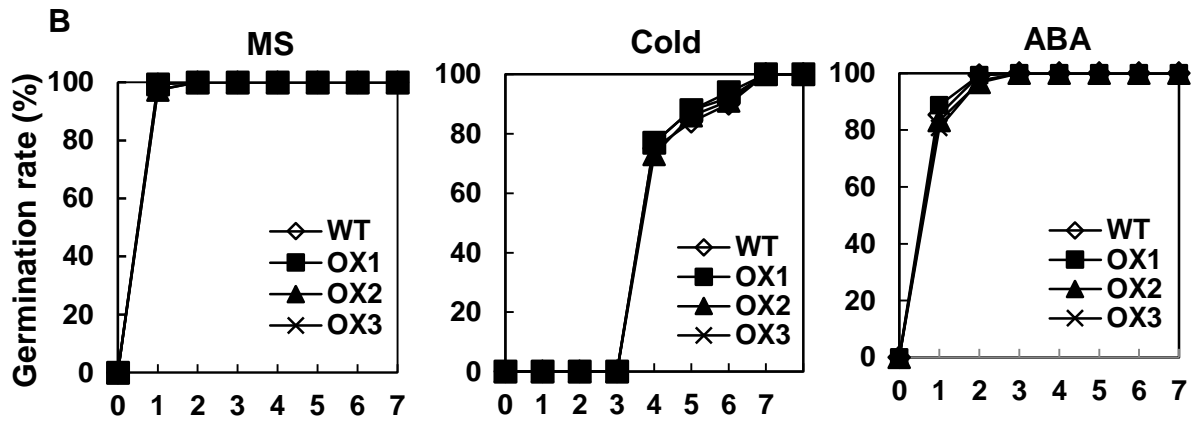

Additional file 1

Supplement: Supplementary file 1 — Confirmation and seed germination of transgenic plants under normal, cold, or ABA conditions. (A) Expression of OsRH58 in three homozygous Arabidopsis lines (OX1, OX2, and OX3) was confirmed by RT-PCR. Actin was used as a loading control. (B) Germination rates of the wild type (WT) and transgenic plants were scored on MS medium or MS medium supplemented with 1 μM ABA at normal temperatures, and on MS medium at 10 °C. (PDF 406 kb) [file 12870_2018_1623_MOESM1_ESM.pdf]

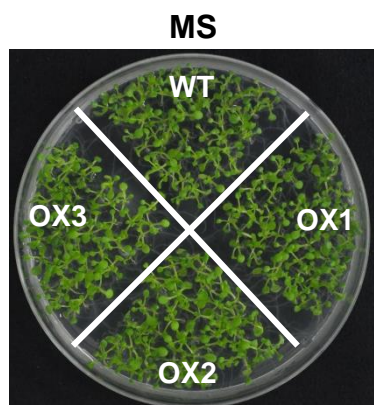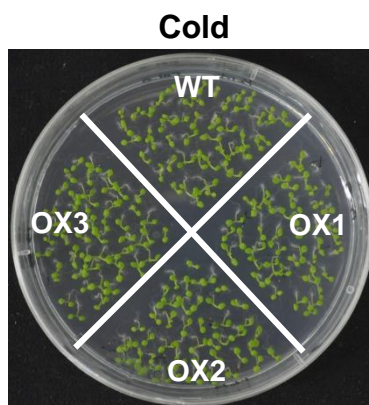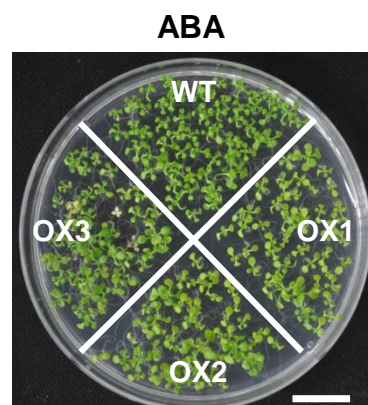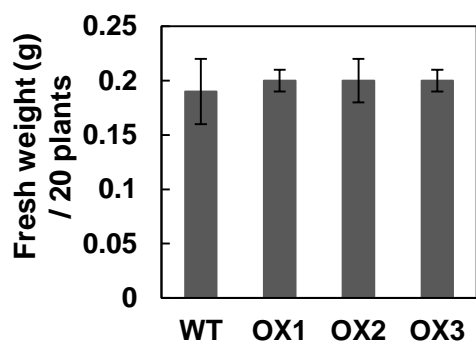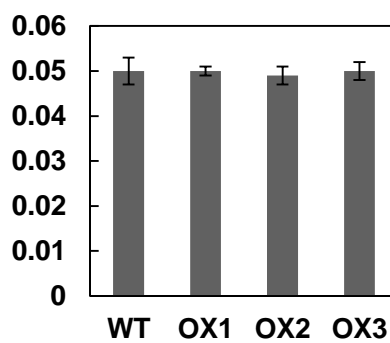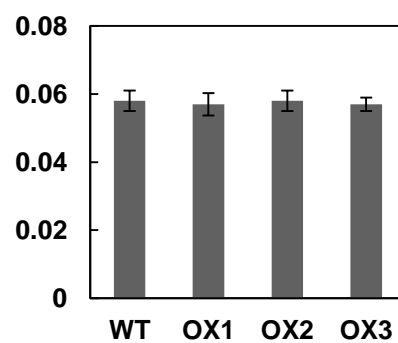

**Additional file 2**

Supplement: Supplementary file 2 — Seedling growth of the wild type and transgenic plants under normal, cold, or ABA conditions. Growth of the wild type (WT) and OsRH58-expressing transgenic Arabidopsis plants (OX1,OX2, and OX3) was analyzed on MS medium or MS medium supplemented with 2 μM ABA at normal temperatures, and on MS medium at 10 °C. The mean and standard error of fresh weight were obtained from three biological replicates. (PDF 408 kb) [file 12870_2018_1623_MOESM2_ESM.pdf]

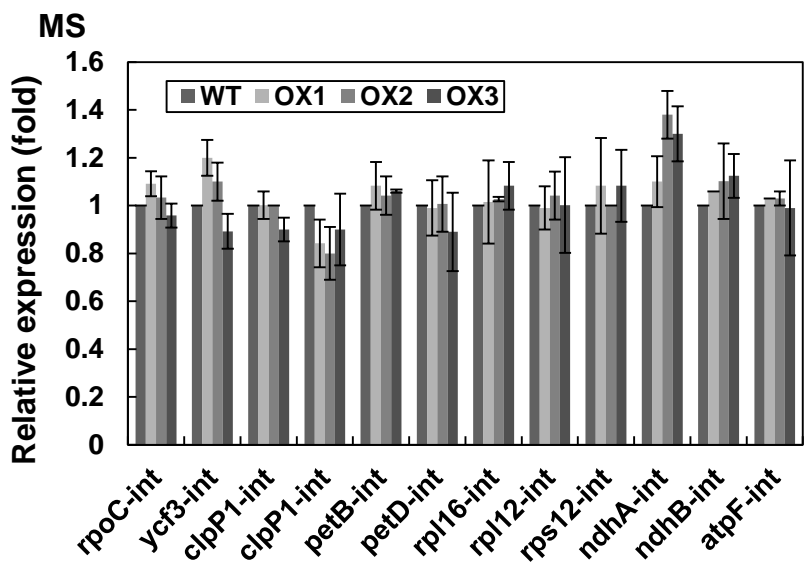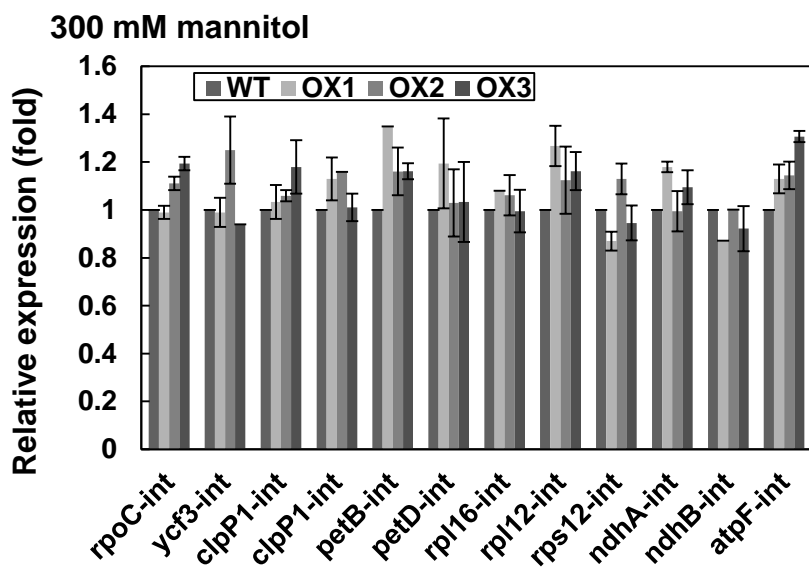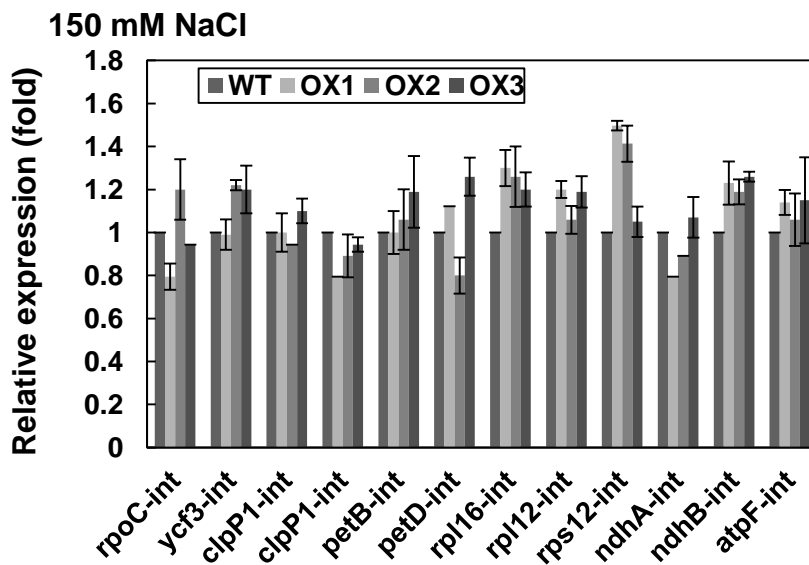

Supplement: Supplementary file 3 — Splicing efficiency of chloroplast intron-containing genes. Total RNA was extracted from 2-week-old wild type (WT) and OsRH58-expressing transgenic Arabidopsis plants (OX1,OX2, and OX3) grown on MS medium or MS medium supplemented with 150 mM NaCl or 300 mM mannitol, and the levels of unspliced and spliced transcripts of each gene were determined by real-time RT-PCR. The mean and standard error were obtained from three biological replicates. (PDF 405 kb) [file 12870_2018_1623_MOESM3_ESM.pdf]

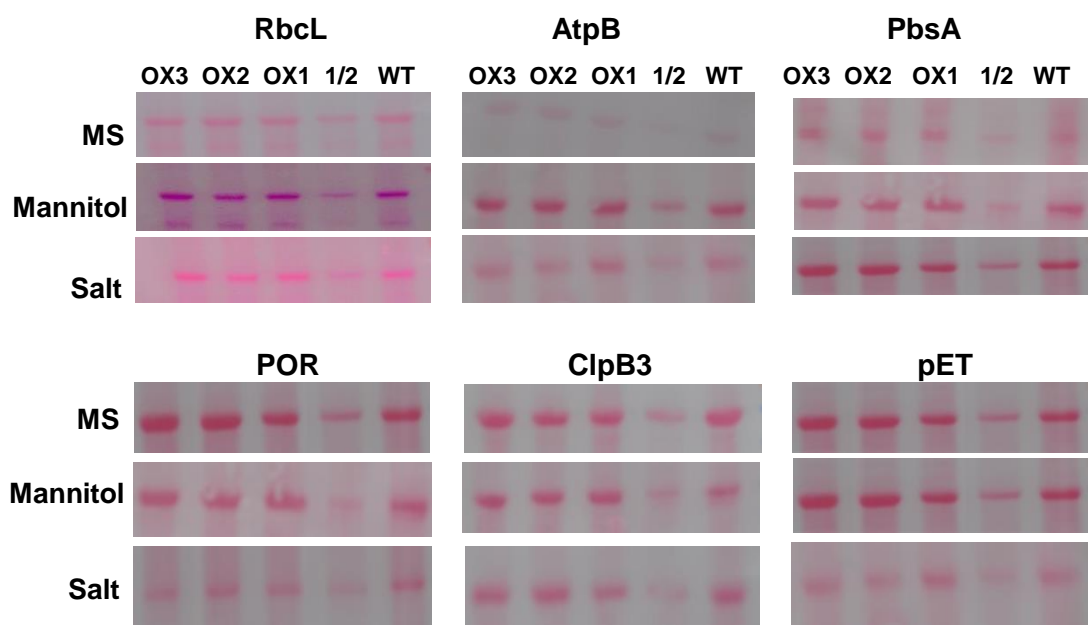

Additional file 4

Supplement: Supplementary file 4 — SDS-PAGE gels showing total proteins in each sample. Total proteins were extracted from 2-week-old wild type (WT) and OsRH58-expressing transgenic Arabidopsis plants (OX1,OX2, and OX3) grown on MS medium or MS medium supplemented with 150 mM NaCl or 300 mM mannitol, and the proteins were separated on SDS-12% PAGE gel, transferred to membrane, and stained with a Ponceau-S. 1/2, half amount of WT protein. (PDF 408 kb) [file 12870_2018_1623_MOESM4_ESM.pdf]
