# Supplementary material for: Rice OsRH58, a chloroplast DEAD-box RNA helicase, improves salt or drought stress tolerance in Arabidopsis by affecting chloroplast translation
Source: BMC Plant Biol. 2019 Jan 9;19:17. doi: 10.1186/s12870-018-1623-8 (PMC6327599; doi:10.1186/s12870-018-1623-8)
Supplement: Supplementary file 5 — List of primers used in RT-PCR and quantitate real-time RT-PCR analysis. (PDF 403 kb) [file 12870_2018_1623_MOESM5_ESM.pdf]

Additional file 5: List of primers used in RT-PCR and quantitate real-time RT-PCR analysis.

Gene	Primers (5' to 3')	
OsRH58	Forward	GAAGAAACCTAGGAGAGCCG
	Reverse	CTTCCTGTCAATTTCTCCTA
MYR	Forward	CAAGACGGACGATCAGGATACGAG
	Reverse	TGAAGGATCAGGTTTCTCCAAATG
MBP	Forward	ATGGGACGATGGATCGACACATG
	Reverse	GTAGTCAACGGAGCAAAGTAAGCC
SAMS	Forward	ACCAAGGCTAACGTTGATTACGAG
	Reverse	GGACACGTACAGGAACCATGGCTC
ISO	Forward	TTGCGGAAGTGCAGACTTGGTGG
	Reverse	TTGCCTCCCTCTGCTTTCTGTCATG
BGU	Forward	GGCTGACCAGAAGGTTGATAGTCG
	Reverse	CCTAGCTGTGTACCCGTCTTGCCA
LEAP	Forward	ATGTGTTCGGCCATACTCAGAAAG
	Reverse	CTCCAGCTAGAGCAATGACGTTGG
SEEDSP	Forward	AACAACCCACAAGGGCAGGAATG
	Reverse	AGCGCTAAGGCGGAGAAGTCTGAG
PHYB	Forward	GTTTCGCAAACTCATTGTTGGAGG
	Reverse	ACTAGGCATTTGCTTTGAAAGG
GA4H	Forward	TTTAGATCGCATCCCATTCACATCC
	Reverse	AGAGCCAATAACGGTGAAACCTTC
ACTIN	Forward	CAGAGCGGGAAATTGTAAGAG
	Reverse	CCTTTCAGGTGGTGCAACGAC
ycf3 ex1	Forward	TTCGGGCATTAGAACGAAAC
ycf3 ex2	Reverse	TCCAATACTCAGCGGCTTG
ycf3 int1	Forward	AGTTGGTTGTCGAGCCGTAT
clpP1 ex1	Forward	TCGAAGTCCTGGAGAAGGAG
clpP1 ex2	Reverse	AATAAGTTGATTCGAGATTTCGGT
clpP1 int1	Forward	GAACCGTATGCACCAAAAGG
petB ex1	Forward	CATTGTATATTTCCGGAATATGAG
petB ex2	Reverse	TATGTTGACATGCGGAGGAA
petB int1	Forward	TCTTGGAGGGGGAGTAACCT
clpP1 ex2	Forward	GTCGGAGGAGCAATTACCAA
clpP1 ex3	Reverse	GTGATGGTTTCGCGAAGTTT
clpP1 int2	Forward	TCATTCTGCGAAATAGAAAAACC
petD ex1	Forward	GAAGAGATAATGGATTATGGGAG
petD ex2	Reverse	GGGTTCCCCGTAATAATTGTG
petD int1	Forward	AAAAATTATCATGTCCGGTTCC
rpl2 ex1	Forward	AAATGGGAAATGCCCTACCT
rpl2 ex2	Reverse	GGACCTCTCCAGAAGGTAAT
rpl2 int1	Forward	GCCGTATGCTTTGGAAGAAG
rpl16 ex1	Forward	CTTTGATATAATTGCTATGCTTAG
rpl16 ex2	Reverse	CCAAATTTTTCCACCACGTC
rpl16 int1	Forward	AAACTCTCACGTTCAGTTCTG
rps16 ex1	Forward	AGCTGTTCTTGTTGGTTGAGC
rps16 ex2	Reverse	TTTTCTCGAGCCGTACGAGG

rps16 int1	Forward	TTCTCGAGCCGTACGAGGCCA
rps12 ex2	Forward	CGTAAAGTTGCCAGAGTACGA
rps12 int2	Reverse	TGTGGAAAGCCGTATTCGAT
rps12 ex3	Forward	TTTGGCTTTTTGACCCCATA
rps12 ex1	Forward	ATCCGAAACGTCACGAAATC
rps12 ex2	Reverse	TCTCACACCGGGTAAATCCT
rps12 int1	Reverse	GGAGCCGTATGAGGTGAAAA
ndhB ex1	Forward	TCATCAATGGACTCCTGACG
ndhB int1	Forward	AGTCTCATGCACGGTTTTGA
ndhB ex2	Reverse	CCAGAAGAAGATGCCATTCA
atpF ex1	Forward	TACTTGGGTCACTGGCCATC
atpF int1	Forward	TTCGGGAAGGGATCATAGAA
atpF ex2	Reverse	GCTCCTTCACGCAGTTCTTC
ndhA ex1	Forward	TTAGGTGGTCTGCGAGCTG
ndhA int1	Forward	AGGCCAAGACCTCATGTACG
ndhA ex2	Reverse	TTGACGCCACAAATTCCAT
